# Inhibitory effects of thymol and carvacrol on heme degradation and oxidative products due to tartrazine: *In silico* and *in vitro* studies

**DOI:** 10.1016/j.heliyon.2024.e24576

**Published:** 2024-01-17

**Authors:** Parvaneh Fakharian, Fereshteh Taghavi, Zahra Kianmehr, Maryam Atashian

**Affiliations:** a Department of Biochemistry, Faculty of Biological Sciences, North Tehran Branch, Islamic Azad University, Tehran, Iran; b Faculty of Biological Sciences, Tarbiat Modares University, Tehran, Iran

**Keywords:** Oxidative stress, Human hemoglobin, Natural antioxidants, Industrial dyes, Human health

## Abstract

The pathology of many diseases arises from oxidative stress and cell destruction. Antioxidant application is one of the most important ways for oxidative stress prevention in the cells and its consequent effects. The present study investigated the natural antioxidants inhibitory effects of thymol and carvacrol on human hemoglobin treated with tartrazine. Purified hemoglobin from human blood samples was treated with tartrazine alone or in combination with mentioned natural antioxidants (thymol and carvacrol). Treated samples were picked up at regular time intervals and changes were followed by UV–visible and fluorescence spectroscopic assays, and circular dichroism spectroscopy (CD). The result of fluorescence spectroscopy revealed that thymol and carvacrol prevented the production of heme-degradation products and advanced glycation end products (AGEs) caused by hemoglobin oxidation with tartrazine. The results of UV–visible and fluorescence spectroscopy revealed the positive effect of these antioxidants on preserving Hb folding, heme, and especially the porphyrin ring surrounding the microenvironment. The results of the circular dichroism (CD) assay showed the protection of alpha helix structure in hemoglobin treated with thymol and carvacrol compared to the control sample. The mentioned antioxidants caused hemoglobin resistance against tartrazine's destructive effect by preventing both heme degradation and glycemic toxins formation and thus reducing the rate of oxidative processes. This matter can be important for various pharmaceutical, health, and cosmetic industries.

## Graphical abstract

**Figure:**
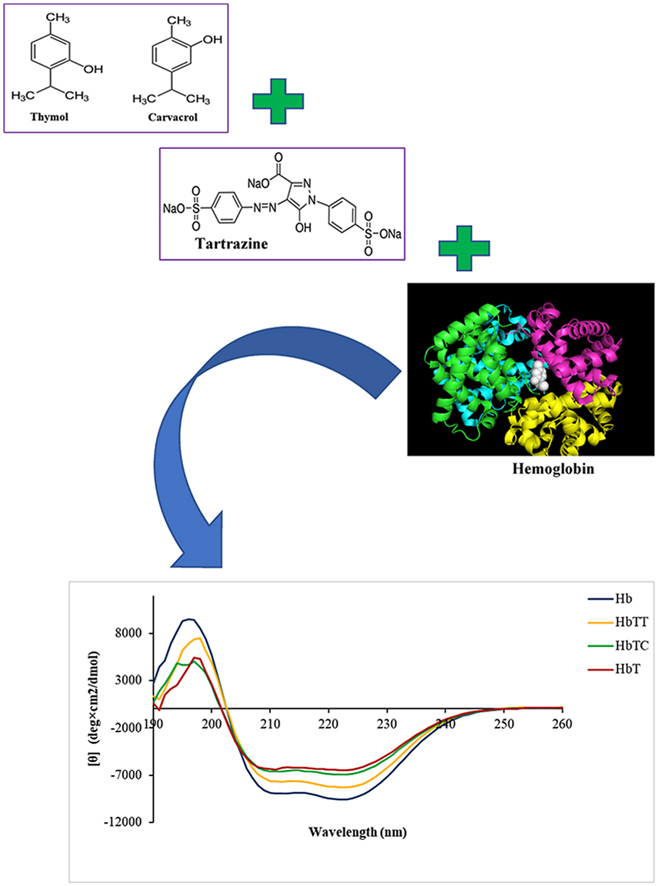

## 1 Introduction

Any disorders in the normal state of oxidation-reduction (oxidative stress) through the production of aggressive species cause structural and functional changes in important bio-macromolecules in the human body, including proteins (especially plasma protein) and nucleic acids [1,2].

In this way, these processes can put human life in danger and induce or accelerate the appearance of various diseases, such as cancer, diabetes, cardiovascular and inflammatory diseases [[3], [4], [5], [6]], Alzheimer's, Atherosclerosis, and diabetes [[7], [8], [9], [10], [11], [12], [13]]. Hemoglobin as a plasma protein, characterized by vital function, especially the oxygen delivery system could be targeted for such oxidative changes. Hemoglobin consists of two alpha and two beta chains (α_2_β_2_); the alpha chain consists of 141 amino acids and the beta chain consists of 146 amino acids. Each alpha and beta polypeptide chain in hemoglobin is attached to a heme prosthetic group [[14], [15], [16], [17]].

One significant specification of hemoglobin is the presence of aromatic amino acids such as tryptophan, tyrosine, and phenylalanine around the porphyrin ring. Hemoglobin contains three tryptophan residues (Trp 15, 37 β and Trp 14α and five tyrosine residues (Tyr 34, 144 β and Tyr 24, 42, 140 α) in each hemoglobin chain dimer, but not all could be titrated [14,[17], [18], [19]]. Hemoglobin plays a vital role in respiratory gas transportation and blood pH regulation [17].

The divalent iron ion in the heterocyclic porphyrin ring determines the hemoglobin oxygen-binding capacity, and a significant interaction occurs between heterologous subunits in hemoglobin quaternary structure [14,15].

The use of antioxidants is an important way of inhibiting oxidative stress in cells and its outcomes. In this regard, medicinal plant extracts as natural products have always been considered for biomedical purposes [[20], [21], [22]].

Flavonoids are a class of antioxidant compounds found in plants bearing antioxidant properties due to hydroxyl groups located in their cyclic structures. Flavonoids, as hydrogen donors and inhibitors of hydroxyl free radicals as well as superoxide, activate antioxidant enzymes and reduce oxidative stress [1,3,4].

Butylated hydroxytoluene (BHT) and butylated hydroxy anisole (BHA) as synthetic antioxidants have been used in industry, but due to their destructive effects, the use of natural antioxidants instead aims to eliminate or at least lower the effect of the chemicals which has received special attention of researchers [3,5,6]. Essential oils of some plants including (thyme) contain a natural monoterpenoid called thymol, bearing antioxidant, antimicrobial, and anti-diabetic properties [3,5,[22], [23], [24]].

**Fig. 1:**
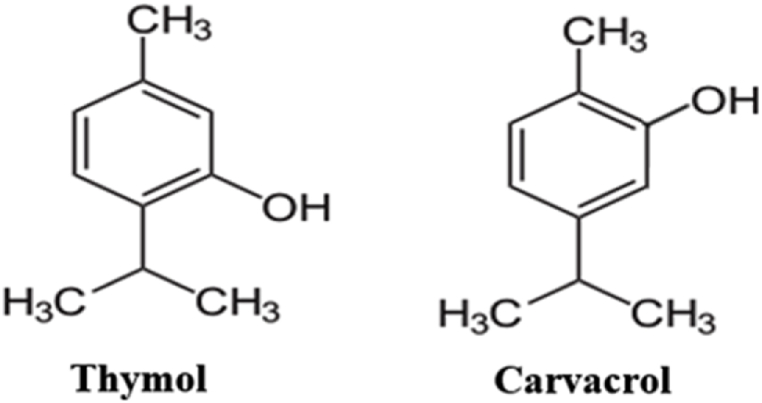
Thymol and carvacrol chemical structure.

Carvacrol considered as a thymol isomer is a natural monoterpenoid that is prepared from *p*-cymene. Carvacrol has been shown to exhibit antioxidant, antitumor [6,22,25], antimicrobial, and flavoring properties [26] (Fig. 1). The presence of hydroxyl groups and double bonds causes to increase antioxidant properties of thymol and carvacrol in phenolic compounds [27].

Esmaili et al. investigated the antioxidant activity of aromatic compounds such as thymol and proved satisfactory redox power of thymol [4].

Aman et al. showed the thymol antioxidant effect as a free radical scavenger and its relevant protective effect on red blood cell hemolysis in normal people as well as diabetic patients. The results showed that thymol could be considered as a traditional (natural) drug [5].

Turkez et al., by using total oxidant stress (TOS) and total antioxidant capacity (TAC) methods, studied the antioxidant/oxidative potentiality of carvacrol and therefore reported that carvacrol increased the TAC levels at concentrations of 50 ‚75–100 mg/L [6].

Also, the study of both carvacrol and thymol antioxidant activity, as a carvacrol isomer, in human neutrophils showed that carvacrol can be suitable as a natural antioxidant in foodstuff and pharmaceutical industries [[28], [29], [30]].

**Fig. 2:**
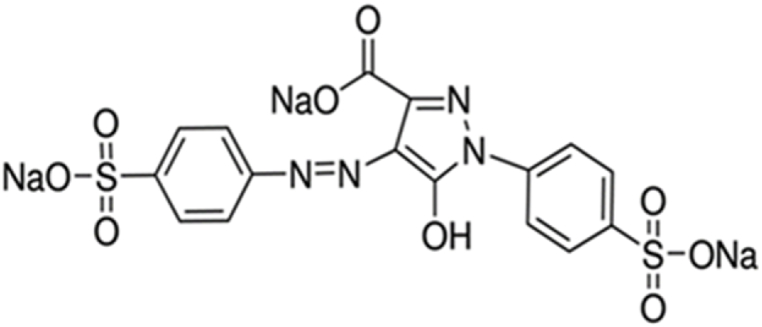
Tartrazine structural formula (Sigma-Aldrich).

Food colors are natural or synthetic additives and are considered important and influential factors in the appearance quality and food marketability [31]. Tartrazine (Yellow No.5, E102) is a synthetic azo benzene dye, widely used in food coloring, medicine, and cosmetics [15,32,33] (Fig. 2). Azo dyes are synthetic compounds characterized by one (mono-azo) or several intramolecular –N=N– bonds [34].

Tartrazine due to its oxidative stress potentiality, in addition to its primary destructive effects, such as accelerating carcinogenesis [[35], [36], [37], [38]], asthma, eczema, urticarial, angioedema, migraine, and hyperactivity in children [39], may also induce other effects.

Kamaljeet et al. [15] and Li et al. [17] have concluded that azo industrial dyes as a toxic and destructive oxidizing agent react with bovine hemoglobin (BHb) and insert significant effects on this protein function.

Basu et al. investigated the tartrazine binding to human hemoglobin on multi-spectroscopic and colorimetric assay basis. This study showed that tartrazine-treated hemoglobin revealed hypochromic changes (shifting to shorter wavelengths as solvent polarity changes) [40].

Considering the phenolic structure of carvacrol and thymol and the possibility of their effect on HB structural changes and their destructive consequent events due to tartrazine, the present study was organized to study important changes by using various spectroscopic methods.

## 2 Material and methods

### 2.1 Material

Tartrazine with a molecular weight of 534.36, granular thymol and solid substance with a purity of 98.5 %, and carvacrol solution with a purity of 98.5 % were purchased from Sigma. Sodium citrate, ammonium sulfate, disodium hydrogen phosphate (Na_2_HPO_4_) and sodium dihydrogen phosphate (NaH_2_PO_4_) were purchased from Merck company. All of substances had analytical grade.

### 2.2 The study strategy

**Fig. 3:**
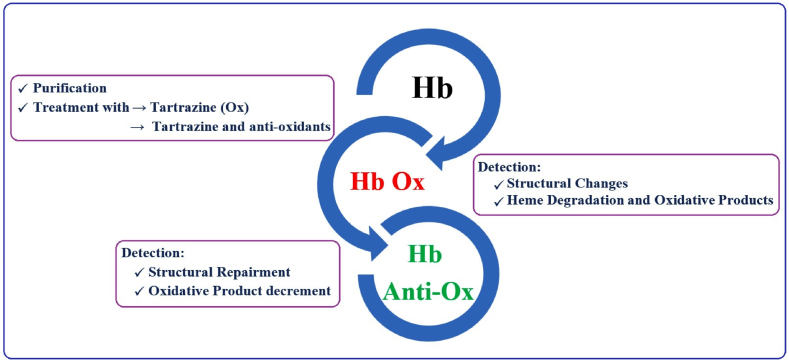
The study strategy.

The present study was conducted based on the strategy mentioned in Fig. 3.

#### 2.2.1 Extraction of hemoglobin from human blood

The blood sample was taken from a healthy non-smoking male without any underlying disease. The following steps were taken to extract hemoglobin from fresh blood [41].

9 mL of fresh blood was first added to 1 mL of 4 % sodium citrate. To separate the serum, the blood sample was centrifuged for 20 min at 3000 rpm (Hitachi RXII series model, made in Japan) and discarded supernatant (serum) afterward.

For suitable red blood cell washing and separation from serum constituents, 0.9 % saline solution (10 times the volume of the sample) was added to the sample and after mixing, it was centrifuged for 15 min at 10,000 rpm and the supernatant was discarded. The washing procedure was repeated two to three times till the supernatant was clear. For re-washing the red blood cells, 0.2 M phosphate buffer (pH 7.4) was added (5 times the volume), and after mixing, then it was centrifuged for 15 min at 5000 rpm, and the supernatant was discarded.

To lyse the red blood cells and separate their hemoglobin content, distilled cold water equivalent to five to nine times the volume of the precipitate was added, and after mixing, it was centrifuged at 15,000 rpm for 15 min. The hemoglobin containing the supernatant was then transferred to a new tube. Considering the volume of the solution, ammonium sulfate was added to the final concentration of 20 % and was gently mixed and left at room temperature (RT) for 15 min. It was then centrifuged at 14,000 rpm for 1 h. Thus, the supernatant (containing hemoglobin) was separated from the cell wall containing residue and dialyzed against 50 mM phosphate buffer (pH 7.4) for 48 h at 4 °C.

Hemoglobin concentration was calculated using the Beer-Lambert equation. The purification of the extracted hemoglobin was confirmed by electrophoresis on 12 % polyacrylamide gel (SDS-PAGE).

#### 2.2.2 Molecular docking studies

For the preliminary investigation of the inhibitory effect of these antioxidants in preventing heme degradation and hemoglobin structural changes, their effective area and possible binding sites on hemoglobin were investigated by molecular docking study.

For this purpose, hemoglobin three-dimensional structure (PDB ID: 1G09) was obtained from the protein database (PDB), and thymol, carvacrol, and tartrazine structures were obtained from ChemSpider site (http://www.chemspider.com).

The molecular connections were checked applying AutoDockTools-1.5.7/Vina from the Scripps Research Institute (http://autodock.scripps.edu/references), and all receptor and the ligand files conversion to PDBQT format was done as well. Then, all water molecules were removed and the Gasteiger charges and polar hydrogen atoms by MGLTools (version 1.5.7) were accepted in the protein file. The dimensions of the grid box were chosen to be large enough with the grid center dimensions at x = 1.127, y = 61.139, and z = 13.0545 and the number of points on 70 × 60 × 70 XYZ with a distance of 0.375 Å. All figures were visualized by PYMOL software and the docking scores representation by the binding free energy (ΔG) was shown via ADVINA.

#### 2.2.3 Hemoglobin treatment with tartrazine, thymol, and carvacrol

The concentration of carvacrol was chosen as 50 μg/mL based on the range of its highest antioxidant activity (50, 75–100 μg/mL) [6] and below its minimum inhibitory concentration (150–400 μg/mL) [42]. Considering the isomerism of carvacrol and thymol and the necessity of comparison, the concentration of thymol was considered equivalent to carvacrol [43].

Therefore, hemoglobin treatment (1000 μg/mL = 15 μM) [44] with tartrazine (534 μg/mL = 1 mM) [35], carvacrol (50 μg/mL = 330 μM) [42], and thymol (50 μg/mL = 330 μM) [43] in different groups was conducted under sterile conditions, and the samples were incubated at 37 °C. Samples were removed from the incubator at 7, 14, 30, and 60-day time intervals. Each sample was then divided into smaller volumes and frozen at once using liquid nitrogen and kept at −70 °C.

#### 2.2.4 The study of hemoglobin structural changes

Hemoglobin structural changes in samples were studied using UV–visible, fluorescence, and circular dichroism spectroscopy (CD) assays.

##### 2.2.4.1 UV–visible spectroscopy

To define the absorption spectrum changes belonging to the prosthetic heme group and hemoglobin structural changes relevant to the amino acid tyrosine absorption, in tartrazine-treated hemoglobin samples alone or with thymol and carvacrol combined, the visible-ultraviolet absorption spectrum was recorded in the range of 200–700 nm using a spectrophotometer (Spectrometer UV–Vis M10). All spectroscopic experiments were conducted in 50 mM phosphate buffer (pH 7.4) at 37 °C and they were repeated three times.

##### 2.2.4.2 Fluorescence spectroscopy

The hemoglobin intrinsic fluorescence emission intensity changes in tartrazine-treated hemoglobin alone or in combination with thymol – carvacrol were measured. For this purpose, hemoglobin (1.25 μM) excitement was measured at 280 nm and the emission intensity was recorded in the range of 300–700 nm in fluorescence spectroscopy (PerkinElmer LS55). Excitation wavelengths at 321 and 460 nm were conducted to trace heme degradation and its induced products. Emission and absorption fission expanse was adjusted to 5 and 10 nm, respectively. The cell length was 1 cm. All experiments were repeated three times at 37 °C.

##### 2.2.4.3 Circular dichroism spectroscopy (CD)

CD spectroscopy functions on basis of the difference between left and right polarized light absorption. Spectra were obtained with a spectropolarimeter (model J-810) in the far ultraviolet region (195–260 nm) which coincides with peptide bond absorption. This analysis aimed to investigate secondary structure changes and their constituent substances in tartrazine-treated hemoglobin alone or in combination with thymol and carvacrol, with respect to control hemoglobin samples on selected days. 50 mM phosphate buffer (pH 7.4) was used to dilute the samples. A protein solution (5 μM) and a quartz cell with a 0.2 cm path length were used.

The residue's mean ellipticity was calculated by the following equation:

[θ]= (θ_obs_ / 10) (MRM/LC)

Where [θ] is the molar ellipticity of residue at wavelength λ (in deg. cm^2^ dmol^−1^), θ_obs_ visible ellipticity in millidegree; MRM is the weight of the average residue; L is the path length in centimeters; and C is the concentration in mg/mL.

The changes in secondary structure elements were analyzed using CDNN.

#### 2.2.5 Statistical computations

All experiments were assayed in triplicate and the graphs are drawn on basis of the mean standard deviation using Excel software.

## 3 Results

### 3.1 Investigation of quality and purity of hemoglobin

**Fig. 4:**
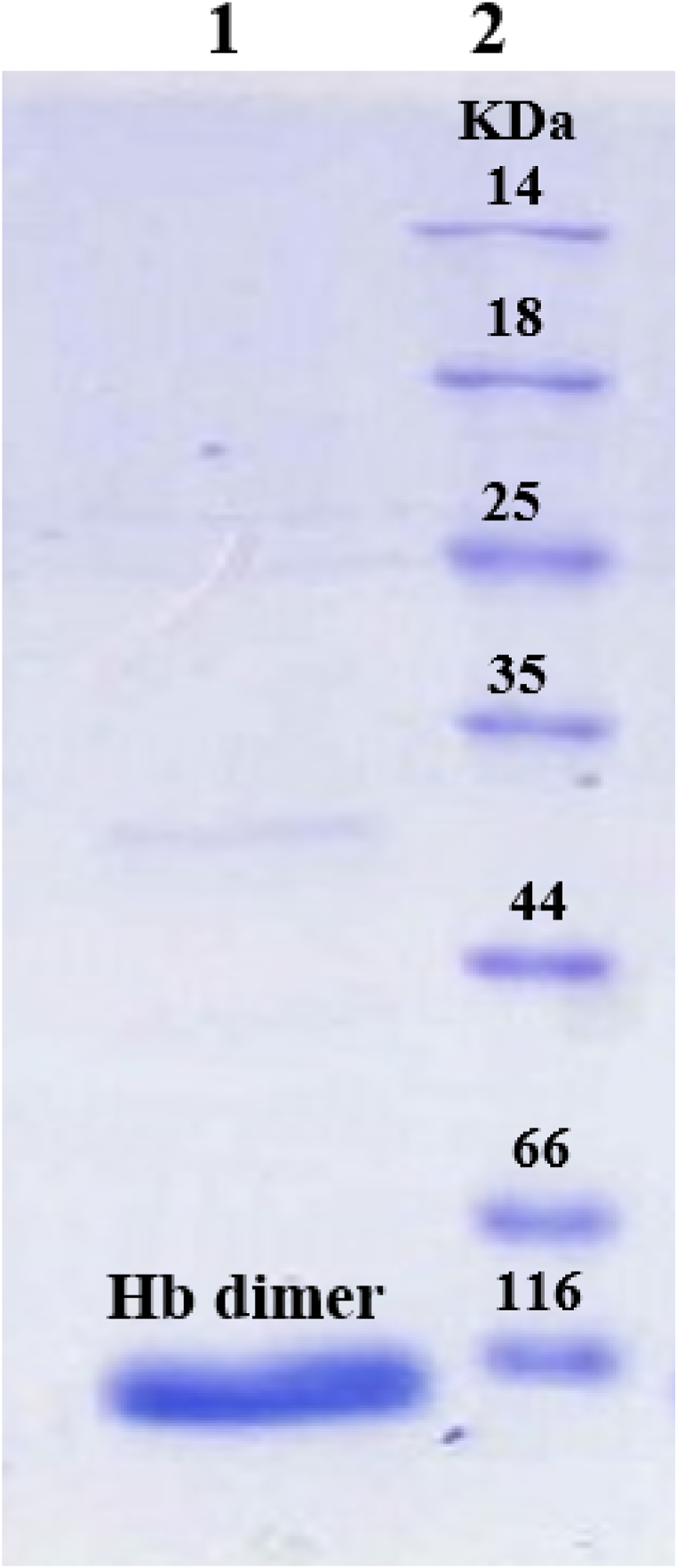
Electrophoresis of extracted hemoglobin on 12 % SDS-PAGE gel. 1)Extracted hemoglobin and 2) Protein marker. The full and unadjusted image of SDS-PAGE gel is provided as supplementary material (S1).

The extracted hemoglobin was electrophoresed on 12 % SDS-PAGE gel. The obtained hemoglobin molecular weight was 64 KDa, and the purity of extracted hemoglobin was confirmed (Fig. 4).

Positioning of hemoglobin dimer sample in the vicinity of 116 KDa and aligning it with the marker protein reveals the optimal position and desirable purification of this protein.

### 3.2 Molecular docking study

**Fig. 5:**
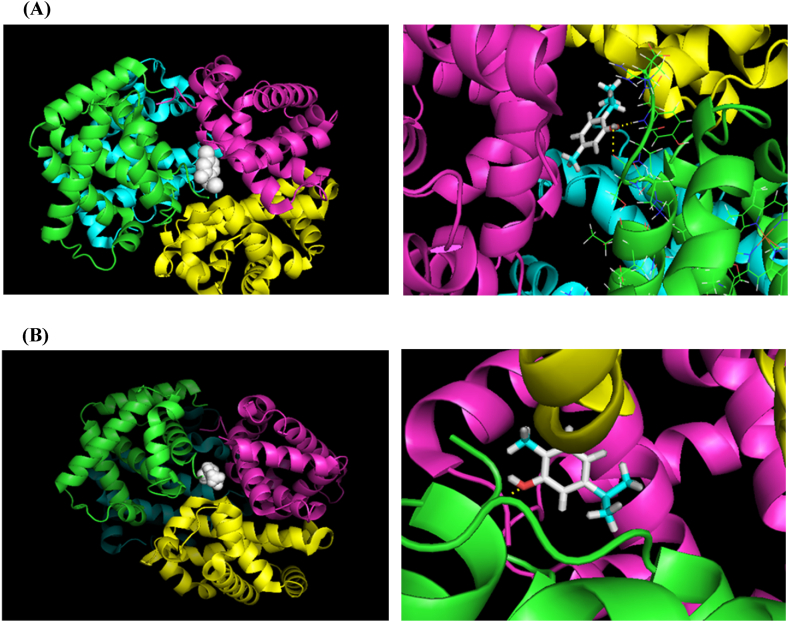
The probable connection sites between human hemoglobin and antioxidants. (A) Hb interaction with thymol; (B) Hb interaction with carvacrol.

The initial investigations of the possibility of research began with the docking study. Fig. 5 referred to the possibility of physical interaction between hemoglobin structure with thymol or carvacrol. In regard to Fig. 5 A, thymol was attracted to hemoglobin central cavity and it made two electrostatic connection points with subdomain Hb-α1 at this region. The binding energy was equal to −6.0 kcal M^−1^. In comparison with thymol, results revealed one connection between carvacrol and Hb-α1 subdomain near to central water cavity (Fig. 5 B). The binding energy was calculated at −6.3 kcal M^−1^.

### 3.3 The effect of antioxidants on toxic products caused by hemoglobin oxidation

#### 3.3.1 Production of heme degradation derivatives

To investigate the presence and development of induced heme degradation products in treated hemoglobin with tartrazine alone or with both tartrazine and one of the antioxidants, the presence of two known fluorophore species possessing a non-protein structure due to non-enzymatic degradation of the heme prosthetic group was studied. The first and second species excitation were studied at 321 nm and 460 nm, respectively.

##### 3.3.1.1 The first induced species from heme degradation

**Fig. 6:**
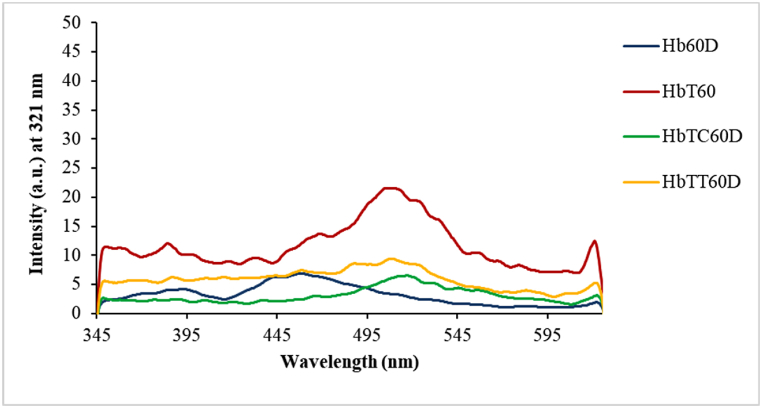
Detection of first species products due to heme group degradation in treated hemoglobin after 60 days. Hb: control hemoglobin, HbT: tartrazine-treated hemoglobin, HbTT: tartrazine and thymol-treated hemoglobin, HbTC: tartrazine and carvacrol-treated hemoglobin. Each range is the mean of the conducted repetitions. 60D represents the sixtieth day after treatment.

To detect the production of the first-induced species from heme degradation, the samples were excited at 321 nm, then investigated relevant fluorescence emission rated at 330–700 nm range. Generation of the first species of fluorophore due to heme degradation in samples on day 60th of treatment revealed an increase in emission peak intensity and a shift of HbT emission peak with respect to the control (Fig. 6). This is an indication of the heme group degradation and the production of the first species resulting from its destruction. Also, examining the effectiveness of the antioxidants (thymol and carvacrol) on inhibiting heme degradation product showed the best effects of carvacrol on decreasing the production of the first species with respect to HbT as the control sample.

##### 3.3.1.2 The second induced species from heme degradation

**Fig. 7:**
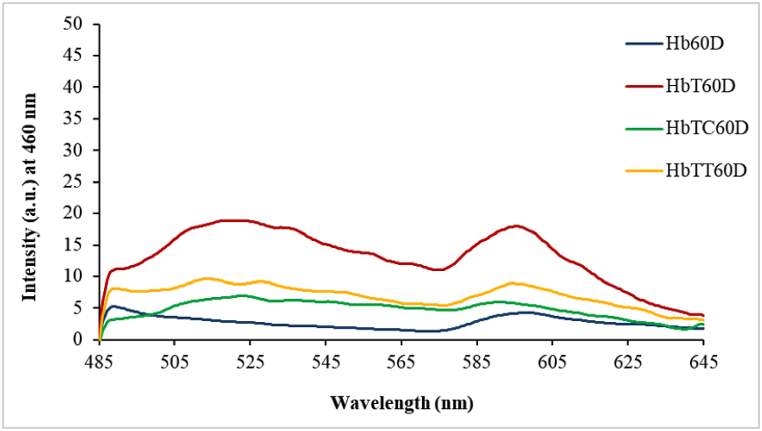
Detection of second species products induced by heme group degradation on treated hemoglobin samples at 60th day. Hb: control hemoglobin, HbT: tartrazine-treated hemoglobin, HbTT: tartrazine and thymol-treated hemoglobin, HbTC: tartrazine and carvacrol-treated hemoglobin. Each range is the mean of conducted repetitions. 60D represents the sixtieth day after treatment.

The detection of second species production due to heme degradation was done by samples exciting at 460 nm. Then their fluorescence emission rate was studied at the 470–700 nm. On the 60th day of treatment (Fig. 7), the peak emission intensity increment of the HbT sample, compared to the control (Hb), demonstrated the generation of second species due to heme degradation with respect to control hemoglobin.

In the evaluation of the effect of treating with carvacrol (HbTC) and thymol (HbTT), a notable reduction on the production of the second species of heme degradation was observed with the superiority of carvacrol, with respect to the tartrazine-treated hemoglobin.

#### 3.3.2 The production of oxidative glyco-toxins

**Fig. 8:**
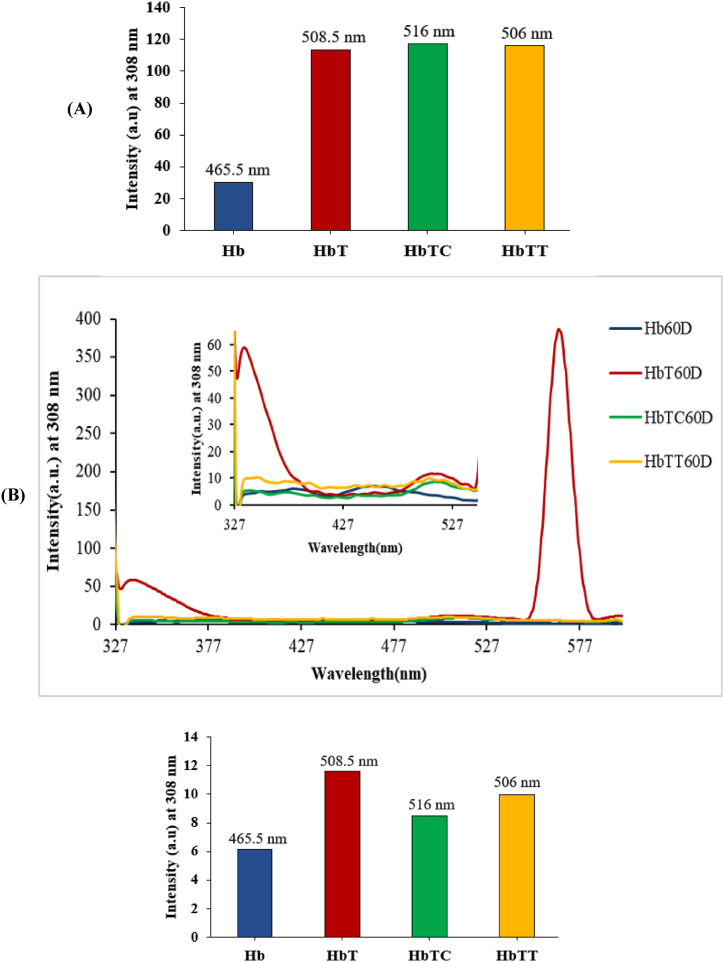
Detecting the emission intensity increment and formation of advanced glycation end products (AGEs) in hemoglobin-treated samples after (A) 30 days and (B) 60 days Hb: control hemoglobin, HbT: tartrazine-treated hemoglobin, HbTT: tartrazine and thymol-treated hemoglobin, HbTC: tartrazine and carvacrol-treated hemoglobin. Each range is the mean of the conducted repetitions. D30 and D60 represent 30 and 60 days after treatment.

To detect the production of advanced glycation end products (AGEs) and evaluate the effects of thymol and carvacrol on preventing these toxins formation, the excitation and the fluorescence emission rate of all Hb samples were measured at 308 nm and 308 nm, respectively. In the 30-day of treatments (Fig. 8A), the emission intensity increment in the HbT sample with respect to the control hemoglobin is an indication of an increase in the production of oxidative AGE products. No significant antioxidant effect was observed on the reduction of AGEs products within the treating time period. Meanwhile, in the sixty-day of treatment (Fig. 8B), the increase in emission intensity appearing as three peaks (at 339, 510, and 567 nm) in the HbT sample compared to the control sample, indicates the production of three species of AGE products. Also, the deletion of two large peaks and emission intensity reduction in the middle peak region respectively by thymol and carvacrol indicated their positive effect on reducing the production of AGE products. No AGE products were seen on the seventh day of treatment.

### 3.4 The effect of antioxidants on the structure of hemoglobin in the presence of tartrazine

#### 3.4.1 Hb structural changes detected by ultraviolet–visible spectroscopy

UV–Vis spectroscopy assay is conducted to study the structure and conformation of biological molecules induced by light interaction (electromagnetic radiation). In this spectroscopy assay, the quantity and status of all presented chromophores in a compound (aromatic amino acids such as tryptophan, tyrosine and phenylalanine) create a specific adsorption pattern in 200–700 nm range.

The absorption spectrum of pure hemoglobin possesses four absorption peaks, two of which are absorption peaks in the globin region at 280 nm and a soret peak at 406 nm region as well as two smaller peaks located in the Q band in the 500–600 nm range. Hemoglobin, as a metal-protein complex, consists of a strong absorption peak sensitive to environmental changes [15,45,46]. The absence of electron positioning amongst the four pyrrole porphyry rings induces changes in adsorption intensity in the peak region because of the hemoglobin absorption spectrum susceptibility to ligand changes bond to heme iron, and these changes could be conducted to study the structure and conformation of the case protein [47].

**Fig. 9:**
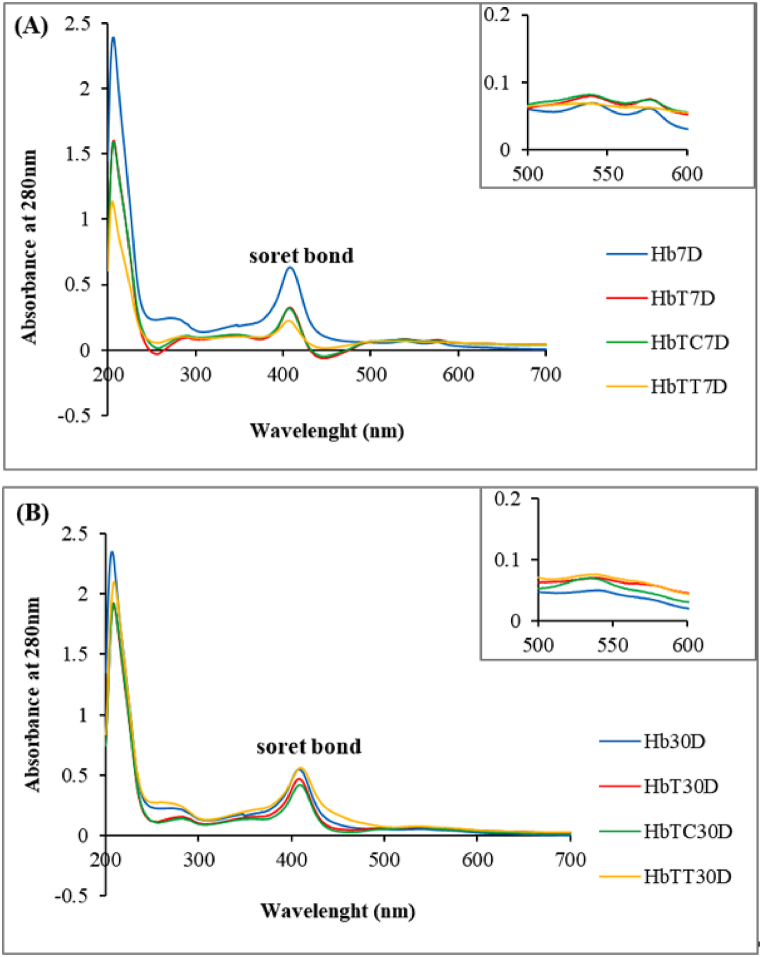
**Absorption spectrum changes in the wavelength range of 200**–**700 nm,** hemoglobin treated samples (A) on the seventh day and (B) on the 30th day. Hb: control hemoglobin, HbT: tartrazine-treated hemoglobin, HbTT: tartrazine and thymol-treated hemoglobin, HbTC: tartrazine and carvacrol-treated hemoglobin. Each range is the mean of repetitions conducted. 7D represents day seven and 30D represents day 30 of treatment.

Absorption spectra of tartrazine-treated hemoglobin alone or combined with thymol and carvacrol, in comparison with control hemoglobin at different time intervals, were measured in a spectrophotometer in 200–700 nm range at room temperature and pH 7.4 (Fig. 9).

On the 7th day (Fig. 9A) and the 30th day (Fig. 9B) of treatment, in HbT, the adsorption intensity decrement was observed at 280 nm region and for the soret peak at 406 nm, while the two small peaks became larger in the 500–600 nm region respect to the control hemoglobin.

These changes were referred to Hb structural changes as a consequence of treating with tartrazine.

On the 7th day of treatment (Fig. 9A), HbTC did not show any significant difference compared to the HbT, except for a slight absorption increment at 280 nm. However, HbTT showed more changes at 280 nm and along 500–600 nm in the form of closer movement to the absorption spectrum of Hb control, but in this sample (HbTT) soret region did not show a positive change.

On the 30th day of treatment (Fig. 9B), an absorption decrement of HbT at 406 nm and 280 nm as well as flattening of two small peaks at 600-500 nm with absorbance increment could be observed due to hemoglobin structural changes with respect to control hemoglobin. The HbTT showed an absorption increment in both 406 nm and 280 nm regions compared to the HbT with greater proximity to control hemoglobin than HbTC. Nevertheless, no positive effects in the 500–600 nm range were observed. Although, HbTC, less than thymol, showed an acceptable effect on one of the smaller peaks only in the range of 600–500 nm, it did not show a positive effect with respect to the control sample in other regions (280 and 406 nm) on the 30th day of treatment. As per these results, it seems that thymol has been more effective than carvacrol in inhibiting the destructive effect of tartrazine on hemoglobin structure on the 7th and 30th days of treatments.

#### 3.4.2 Hb structural changes detected by intrinsic fluorescence spectroscopy

Fluorescence spectroscopy principles are based on emission phenomenon and conducted to investigate the conformational changes in protein tertiary structure due to its intrinsic fluorophore's presence within a protein (i.e., aromatic amino acids such as tryptophan, phenylalanine, and tyrosine). This technique was also used to assess the resulting products of heme destruction, glycation toxins, and intermediates due to their intrinsic emission.

**Fig. 10:**
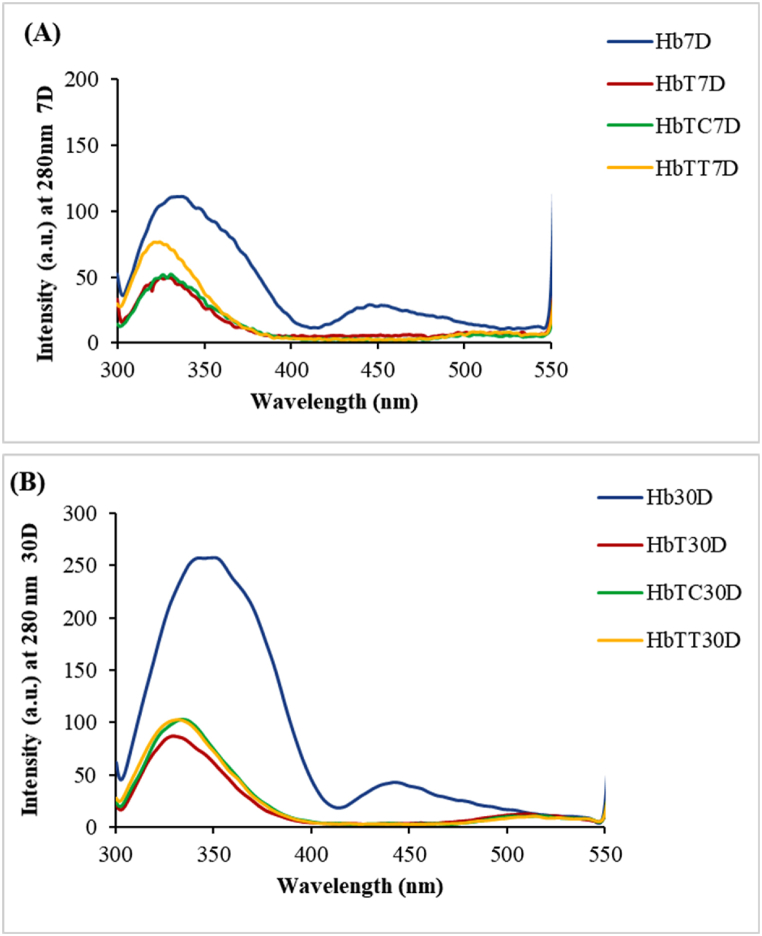
**Detecting the hemoglobin intrinsic fluorescence of treated samples at the excitation wavelength of 280 nm.** (A) On the seventh day and (B) on the thirtieth day. Hb: control hemoglobin, HbT: tartrazine-treated hemoglobin, HbTT: tartrazine and thymol-treated hemoglobin, HbTC: tartrazine and carvacrol-treated hemoglobin. Each range is the mean of the conducted repetitions. 7D represents day seven and 30D represents day 30 after treatment.

As indicated in Fig. 10, a sharp decrease in emission intensity was observed in the HbT in the 7th and 30th day of treatments, due to the hemoglobin tertiary structure changes compared to the control sample (Hb). Emission intensity decrement is an indication of the hemoglobin structure unfolding induced by tartrazine treatment.

In the seventh treatment day, the presence of thymol antioxidants in the hemoglobin treatment medium caused to increase the hemoglobin emission intensity and its relevant spectrum was getting closer to the emission spectrum of the control sample (Fig. 10 A). This obviously indicated the thymol inhibitory effect on the hemoglobin tertiary structure changes as well as its unfolding in presence of tartrazine.

Meanwhile, during seven days of treatment, carvacrol did not show any promising effect. This result is in agreement with UV–Visible spectroscopy results.

Results on the 30th day of treatment revealed a protective effect of thymol due to increased emission intensity of HbT incubated with thymol and also its inhibitory effect on hemoglobin unfolding compared to HbT alone. Besides, on the 30th day of treatment, the carvacrol inhibitory effect seemed to be equal to thymol on unfolding prevention of HbT (Fig. 10 B). Thus, it seems that carvacrol takes longer to exhibit the desirable effects on hemoglobin structure than thymol.

#### 3.4.3 Circular dichroism spectroscopy (CD) study

Spectra of all samples in the Far-UV-CD region at 190–260 nm, in the region of α and β structures, were recorded.

**Fig. 11:**
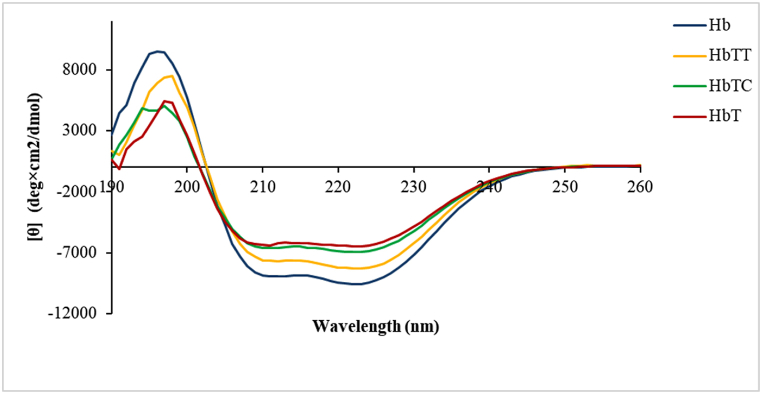
Circular dichroism spectra (CDs) in hemoglobin-treated samples. Hb: Control hemoglobin, HbT: Tartrazine-treated hemoglobin, HbTT: Tartrazine-thymol treated hemoglobin and, HbTC: Tartrazine- Carvacrol treated hemoglobin.

Studies were conducted on the changes in the secondary structures of human control hemoglobin sample as well as tartrazine-, thymol-, and carvacrol-treated hemoglobin simultaneously, and data analysis was performed based on a comparison of molar ellipticity at 220 nm wavelength. The results reveal clear changes in molar ellipticity samples of HbT, HbTT, and HbTC with respect to the control sample and HbT. As per obtained results from the CD assay (Fig. 11), it was found that the molar ellipticity diagram of the control (Hb) is in its lowest position compared to the other samples after seven days. This characterizes as the highest or most negative molar ellipticity in the control sample of hemoglobin. However, HbT was allocated at the lowest molar ellipticity due to its position in the highest region of the graph compared to samples treated with antioxidants and the control sample, indicating a change in the protein secondary structure and its elements due to tartrazine. Compared with the control Hb and HbT, relevant spectra of simultaneous treatment of hemoglobin and tartrazine with carvacrol (HbTC) or thymol (HbTT), were located below the HbT, respectively, in such a way that HbTC spectrum was closer to the HbT and HbTT spectra in the vicinity of Hb. This meant that both antioxidants had an effect on improving the hemoglobin secondary structure and that thymol exhibited a better effect than carvacrol. The result was in agreement with UV–Vis and fluorescence spectroscopy.

Analysis of the secondary structure of the control hemoglobin treated with tartrazine alone or in combination with carvacrol and thymol (Table 1) revealed that maximum changes of alpha helix into the beta structure occurred in HbT samples, while HbTC, HbTT, and Hb showed less structural changes.

**Table 1:** Percentage of secondary structures in treated hemoglobin samples.

Sample	α- Helix	β -Sheet		β -Turn	Random-coil
		Parallel	Anti-parallel		
**Hb 7D**	28.25 ± 0.24	10.42 ± 0.26	7.72 ± 0.50	16.13 ± 0.03	37.47 ± 0.32
**HbT 7D**	21.93 ± 0.20	8.5 ± 0.10	21.3 ± 0.17	15.21 ± 0.02	33.54 ± 0.07
**HbTC 7D**	22.57 ± 0.10	8.95 ± 0.21	19.1 ± 0.41	15.08 ± 0.35	34.27 ± 0.12
**HbTT 7D**	25.02 ± 0.03	10.29 ± 0.15	12.22 ± 0.22	15.39 ± 0.21	37.15 ± 0.25

HbT secondary structure elements consisted of the maximum percentage of beta structure (29.8 % of parallel and antiparallel) while the content of alpha structure decremented to 21.93 % compared with the control sample, hloding 18.2 % of beta structure and 28.3 % of alpha-helix. This matter revealed the tartrazine destructive effect on two important microstructures of hemoglobin protein by increasing the beta sheets content and consequently increasing the hydrophobic regions at the protein surface as well as decreasing alpha helical structures and therefore protein stability [17,40]. These results were in agreement with what was obtained through fluorescence spectroscopy assay and also CD results.

The analysis results showed a slight increment in alpha structure in HbTC (22.57 %) and a slight decrement in beta structure (28.05 %) with respect to HbT sample. However, in HbTT (containing 25.02 % alpha structure and 22.51 % beta structure) more structural improvements (alpha structure increment and beta structure decrement) were observed than in HbT and HbTC and therefore showed a more desired effect on maintaining Hb secondary structure.

## 4 Discussion

Regarding to the capability of natural antioxidants to reduce oxidative stress or its prevention on the structural changes of hemoglobin (its aromatic amino acids as well as its internal prosthetic heme group), this study aimed to investigate the molecular effects of some antioxidants in protecting hemoglobin against degradation and toxic intermediate production caused by tartrazine treatment.

Choosing the best antioxidants and the possibility of their interaction with hemoglobin was done by *in silico* studies. Fortunately, among our antioxidants, carvacrol and thymol showed notable effects and so both of them were chosen.

The molecular docking results showed the tendency of our antioxidants to interact with hemoglobin around the binding regions of tartrazine. In this way, the possibility of further investigations with more techniques was provided. Ligand/receptor physical interactions are an important process that docking studies allow to observe virtually to investigate the non-covalent binding states between them. For the primary investigation of probable interaction between hemoglobin and thymol or carvacrol and its effects on hemoglobin oxidation, docking was the first study that was performed. Regarding to the docking results (Fig. 4), there were noncovalent interactions between hemoglobin and these antioxidants in which thymol revealed two polar connections with subdomain α1 (via Ser138 and Lys 140) and carvacrol showed one polar connection with subdomain α1 (via Ser138). It is worth mentioning that the Hb central water cavity (in the vicinity of β1 and α1, α2 domains) was the main interaction site with tartrazine [17] and these antioxidants. The binding energies of docked complexes were calculated as −8.8, −6.0, and −6.3 kcal M^−1^ for tartrazine, thymol, and carvacrol, respectively. Therefore, according to the binding energies of thymol and carvacrol, they show a favorable tendency to bind to the hemoglobin central cavity; however, the tendency of tartrazine for this type of binding was higher than thymol and carvacrol.

Though, it seemed that the usage of carvacrol or thymol did not interfere with the binding of tartrazine from point of interaction site, but with regards to their spatial conformation, they can produce steric hindrance for electrostatic binding of tartrazine to Hb and make inhibitory effects for this interaction.

It is worth mentioning that, since carvacrol and thymol have the maximum excitation and emission wavelength of 278 nm/306 nm and 276 nm/304 nm, respectively [48,49], and tartrazine has an excitation range of 310–400 nm and an emission peak at 569 nm [50]. It is noteworthy that these peaks are far from hemoglobin emission peak for better comparing the effect of these two antioxidants on the possible inhibition of hemoglobin degradation caused by tartrazine. To do so, the fresh hemoglobin and hemoglobin treated with tartrazine were selected as controls, and treated Hb samples with both tartrazine and each of the two antioxidants were compared with the control samples.

To follow the actual realization of the interaction predicted in docking studies, fluorescence, UV–Visible and Circular dichroism spectroscopies were applied to detect Hb toxic derivatives and their structural changes. UV–Visible spectroscopy showed an absorption peak decrement in the HbT at 280 nm corresponding to the amino acid tyrosine and 406 nm corresponding to the soret peak region with respect to the control. This decrement indicates the interaction between protein and dye which leads to protein structural changes causing the exposure of aromatic amino acids located in the internal hydrophobic region to the solvent and heme environment perturbation in the presence of dye, which is in agreement with previous research [40].

In addition, not only does UV uptake by chromophores intensively depend on the environmental polarity but also the absorption spectrum of the prosthetic heme group in hemoglobin is highly sensitive to environmental changes, oxidation status, and ligand binding. Consequent changes in the soret peak due to exposing aromatic amino acids in the hydrophobic pocket with a polar solvent followed by the opening of buried hydrophobic surfaces within the ligand-hemoglobin complex and replacement with electrostatic residues are all reflections of protein structural changes. Our result is in agreement with research which showed the decrement in absorption intensity of tartrazine compound with human and bovine hemoglobins and induced changes in their structures due to the exposure of aromatic amino acids located in hydrophobic pockets in the protein backbone with polar solvent and alpha helix decrement in the secondary structure [17,40].

The present study also showed that the tartrazine binding to hemoglobin causes hemoglobin structural changes in the Q band region and also increases absorption in these regions compared to control Hb. The results also showed stronger inhibitory effect of thymol compared to carvacrol against hemoglobin structural changes with tartrazine on days 7 (280 nm and Q band) and 30 (280 nm, 406 nm, and Q band), respectively. It also indicated that thymol in comparison with carvacrol increases structural resistance of hemoglobin in both treatment times, specifically in peptide bonds regions (about 280 nm) and soret peak (about 406 nm). It is worth mentioning that the redshift incidence at 406 nm in the soret peak region of 30-day tartrazine-thymol treated hemoglobin could be due to axochromism and changes in solvent polarity, which causes absorption at longer wavelengths.

This event can also be due to the chromophores surrounded by the solvent and their interaction with the solvent molecules and the change in energy levels of the chromophore molecular balances and solvent polarity decrement due to the structure of tartrazine dye and thymol, which causes max ʎ to increases and tend towards higher wavelengths. It is shown that the existence of oxidizing agent causes microenvironment changes surrounding the heme ring, especially in the Q band position due to polarity and environmental pH changes. This event causes the heme ring to deteriorate and open, followed by protonation of the amino-terminal of the amino acids located inside the heme ring such as histidine [46,51]. This is an indication of hemoglobin quaternary structure change and the oxygen releasing from hemoglobin subunits.

Our present study revealed an intensity increment of absorption spectrum in the Q band region in HbT compared to control hemoglobin at both treatment times, indicating structural changes induced by heme destruction, the Q band termination, and the possibility of oxygen pressure changing as a result of dye-protein complex formation. Meanwhile, HbT peak after 30 days of treatment gradually disappeared and showed absorption increment with respect to HbT (7D). Also, HbT (30D) revealed higher absorbance and blueshift compared to control HbT (30D). This could be relevant to the shift to a high-polarity environment resulting in a slight shift to a lower wavelength with a high absorption ratio (hypochromic shift) [52]. Based on our results, the presence of antioxidants (especially thymol) prevents changes in the environmental condition and effectively maintains hemoglobin structure. None of the studied showed positive effects antioxidants in the small Q bands region after 7 and 30 days of treatment.

In an investigation of intrinsic fluorescence emission spectrum changes, a fluorescence spectroscopy assay was conducted to detect products induced by heme degradation and other oxidative products. Our fluorescence investigation of tartrazine's effect on heme degradation in all samples (Hb, HbT, HbTT, and HbTC) revealed the production of two non-protein degradation species and their alteration due to presence of antioxidants. These fluorophore species were induced from the degradation of the heme prosthetic group which was detected at 321 nm and 460 nm in all tartrazine-treated hemoglobin. This matter confirmed that the tartrazine degraded heme and created two types of heme degradation products: one at 460 nm, with respect to the Hb control, and one specie at 321 nm. However, both antioxidants exhibited a significant reduction of two species, resulted from heme degradation, at both wavelengths of 321 nm and 460 nm. In this regard, carvacrol seems to be more effective than thymol in reducing the production of these products.

On the other hand, previous researches revealed that proteins oxidation is an extremely destructive process and could lead to the proliferation of advanced glycation end products (AGEs) and other related toxins by a non-enzymatic oxidation reaction (Millard reaction pathway) [8,53,54].

A group of researchers also showed that AGEs are complex and heterogeneous molecules inducing significant and fundamental changes in the proteins' physiochemical properties, including a significant increment in molecular weight, the ability of aggregation and cross-link formation, hardness, and strength increment in proteolytic digestion, the efficient reduction of proteolysis and damaged (oxidized) proteins aggregation. These could induce cell dysfunction or integrity loss and disruption of protein structure coordination [7,8,[55], [56], [57], [58]]. The triggering of the Maillard reaction by oxidative products was reported before [59].

The study of AGEs formation after 60 days of treatment showed the intrinsic fluorescence emission intensity increment in tartrazine-treated hemoglobin (HbT) at excitation wavelength 308 nm. This matter could obviously be referred to the initiation of the Maillard reaction and formation of glyco-toxins production (AGEs) with respect to the control sample. Interestingly, the presence of three obvious peaks in HbT were indicative of the simultaneous production of three distinct species of AGE products at examined excitation wavelengths. Moreover, the results showed the positive inhibitory effects of both antioxidants in tartrazine-treated hemoglobin incubated with each of these them (HbTC or HbTT) with respect to the HbT after 60 days. Thus, it seems that AGEs compounds can be controlled by the examined antioxidants. This might be due to their anti-oxidative capacity to inhibit the produced reactive species in the hemoglobin oxidation process or create a steric hindrance for the binding of tartrazine to hemoglobin. It is worth mentioning that the mechanism of this product can be followed by Hb structural changes.

Researchers report that tetrameric hemoglobin contains six Trp and Tyr residues which have an important role in hemoglobin structure [14,16,17,60]. For example, Tyrα-140 and Tyrβ-145 play an important role in the hemoglobin's structure and function by the formation of switch and hinge contacts [61]. Therefore, any changes in the exposure of these amino acids can be the reason for structural changes leading to the production of mentioned toxins, and fluorescence spectroscopy can trap their changes due to conformational alteration.

Based on our fluorescence spectroscopy results, the emission peak decrement of HbT compared to the Hb control indicates hemoglobin conformational changes and tertiary structure unfolding. Also, it seems that tartrazine acts as a quenching agent for hemoglobin with a hypochromic effect. The results of Hb structural changes, especially around the heme ring, are in agreement with the results of UV–Vis spectroscopy and also the previous research [17,40]. Also, present studies on spectroscopic emissions showed that tartrazine-treated hemoglobin (HbT) after both seven and thirty days of treatment caused Hb unfolding compared to the Hb control. However, the present study showed that the presence of thymol or carvacrol increased the emission intensity to similar values of Hb control in a protective mode. This effect increased hemoglobin resistance against tartrazine's destructive effects and structural unfolding. Also, after seven days of treatment, the thymol protective effect was more than carvacrol, and after thirty days of treatment, the effects of both antioxidants were similar. Thus, it seems that carvacrol, compared to thymol, started the positive effects on hemoglobin structure maintenance in a slow manner. With respect to the UV and fluorescence assay results, it seems that the conformational protection performed by thymol and carvacrol antioxidants preserved the heme structure and porphyrin ring from exposure to the solvent and further degradation.

Another method that can show the protective effect of antioxidants on hemoglobin structure is the study of changes of Hb secondary structural elements by CD.

In this regard, the alterations of hemoglobin secondary structures were investigated in the presence of tartrazine, thymol, and carvacrol using the CD technique in the Far-UV CD region. The results clearly showed stability decrement in the structure of the tartrazine-treated hemoglobin (HbT) due to the decrease in the alpha helical content and also β-sheet content increment bringing the hydrophobic regions to the hemoglobin surface compared to the control sample (Hb).

These results are in agreement with the finding of Li et al. [17]: when comparing the two antioxidants thymol and carvacrol, the outstanding effect of thymol in HbTT on reducing molar ellipticity and structural stability increment was more evident by increasing the alpha helix structure and making the HbTT spectrum closer to the Hb control.

Shazia et al. [5] also showed that thymol, because of its higher phenolic contents, demonstrates free radical scavenging activity and ferric iron (Fe^+3^) reducing power. In addition, due to its ability to provide protection against Reactive Oxygen Species (ROS) formation (metabolism toxic by-products), thymol can be used to curb dangerous diseases which threaten human health. So, it seems that thymol, by possessing high phenolic content, has more potential than carvacrol to inhibit hemoglobin unfolding while maintaining its functional structure.

## 5 Conclusion

The present study focused on the effect of natural antioxidants on hemoglobin changes (one of the most important proteins in the body) which led to the production of destructive products. The findings revealed that thymol and carvacrol can prevent both the production of toxins caused by heme degradation and the oxidation processes started by tartrazine as an industrial dye. Also, it was shown that this inhibitory effect of thymol and carvacrol is related to their inhibitory effect on heme destruction and porphyrin ring degradation. This inhibition is actually due to their preservation effect on the Hb tertiary and secondary structural elements (alpha structures and beta structure) which prevents the exposure of the heme structure and inhibits oxidative processes. Regarding to the results and by considering the antimicrobial properties of the two mentioned antioxidants, replacing them instead of preservatives and using them along with industrial colors can be a great help in maintaining the products’ health of various food, pharmaceutical, and health industries. It is obvious that such assessments are not limited to the range of industrial dyes and include many widely-used additives in various industries. Also, the identification and application of other natural antioxidants can establish important research lines for more studies.

## Funding

This research received no external funding.

## Data availability

Data will be made available on request.

## CRediT authorship contribution statement

**Parvaneh Fakharian:** Writing – review & editing, Writing – original draft, Validation, Resources, Methodology, Investigation, Funding acquisition, Data curation. **Fereshteh Taghavi:** Writing – review & editing, Writing – original draft, Validation, Methodology, Investigation, Formal analysis, Data curation, Conceptualization. **Zahra Kianmehr:** Writing – review & editing, Validation, Methodology, Investigation, Data curation, Conceptualization. **Maryam Atashian:** Writing – original draft, Methodology, Investigation, Funding acquisition.

## Declaration of competing interest

The authors declare that they have no known competing financial interests or personal relationships that could have appeared to influence the work reported in this paper.
